# Assessing the feasibility of a classroom-based visual attention training program targeting academics for students with extremely low IQ

**DOI:** 10.1186/s40814-021-00879-z

**Published:** 2021-07-30

**Authors:** Catherine Archambault, Domenico Tullo, Emma Clark, Jocelyn Faubert, Armando Bertone

**Affiliations:** 1 Department of Educational and Counselling Psychology, McGill University, 3700 McTavish St, Montréal, QC H3A 1Y2 Canada; 2 École D’Optométrie, Université de Montréal, Montreal, Canada

**Keywords:** Feasibility, Visual attention training, Adolescents, Intellectual disability academic achievement

## Abstract

**Background:**

This feasibility study investigated the viability of implementing a cognitive-based training program (NeuroTracker) and assessing its potential effects on academic performance for adolescents with extremely low IQ.

**Methods:**

Twenty-six adolescents aged between 11 and 16 years with a Wechsler-based IQs in the extremely low range (*M*_IQ_ = 56.00, *SD*_IQ_ = 13.89) completed 15 training sessions on either the NeuroTracker or an active control task; math and reading performance were assessed using clinically validated instruments before and after training. Recruitment and retention rates, adherence, and properties of the academic measures were assessed.

**Results:**

All recruited participants completed 15 training sessions within a 6-week period. Eighty-three percent of participants meeting initial inclusion criteria completed all stages of the study from baseline to post-intervention assessments. Some limitations of the academic measures were identified.

**Conclusions:**

Results suggest that implementing NeuroTracker as a classroom-based intervention and using clinically validated outcome measures is feasible with this population.

## Key messages regarding feasibility

- What uncertainties existed regarding the feasibility?

Prior to conducting this study, it remained unclear whether cognitive training was feasible for children and adolescents with cognitive capability in the extremely low range (i.e., between 2 and 3 standard deviations below the population mean) and in the classroom setting.

- What are the key feasibility findings?

The findings demonstrated that all participants that met inclusionary criteria were able to progress through the arms of the randomized controlled trial assessing the feasibility of the cognitive training program. Furthermore, the findings highlighted that individuals were able to interact with the NeuroTracker attention training paradigm.

- What are the implications of the feasibility findings for the design of the main study?

The implications of the feasibility findings for the design of the study are twofold. For one, the findings suggest that cognitive training is feasible with this population and can be administered in classroom setting. Second, the findings from this study provide recommendations on the appropriateness of outcomes measures.

## Background

Cognitive training involves repeated practice on a domain-specific, computer-based cognitive task that has demonstrated the potential to improve cognitive functioning across a spectrum of domains [1]. This approach has resulted in promising findings for enhancing cognition for neurotypically developing populations [2] and suggested to be used as an alternative or supplemental treatment approach for individuals diagnosed with neurodevelopmental conditions [3, 4]. Cognitive training studies have demonstrated its benefits ranging from improved performance on similar cognitive tasks (i.e., near-transfer) to improved academics (i.e., far transfer) [5–7].

Attention is one cognitive construct that is significantly related to academic achievement [8]. The relationship between attention and academics is evidenced by the association of attentional capability to reading and math achievement in both typically developing [9] and clinical populations [10]. Thus, previous research has attempted to improve reading and mathematics achievement for learners with developmental disabilities via attention-based cognitive training programs, yielding promising outcomes [5, 11].

While a significant body of research and reviews support the validity of these attention-based cognitive training programs [3, 4], research discounting the benefits of this approach also exists [12]. As part of the contentious debate throughout the field, recent reviews have pointed to the lack of methodological standards as the source of inconsistent results [1, 12, 13]. Green et al. [1] suggest that feasibility studies are a critical step to successfully design, execute, and evaluate the validity of a cognitive training program prior to testing efficacy and/or effectiveness. Although feasibility studies are scarce in comparison to efficacy and effectiveness trials in cognitive training research, they can provide valuable information about the implementation and viability of a program. This information is especially valuable in the case of populations with significant cognitive deficits, such as individuals with intellectual functioning below the average range, a population typically excluded from this area of research.

The exclusion of participants with intellectual challenges in most studies (e.g., [14–16]) presents an area of opportunity to investigate the appropriateness of cognitive training for this population. To our knowledge, cognitive-based intervention protocols for adolescents with extremely low intellectual functioning, an underserved portion of the student population, are scarce. Since these learners often present comorbid attention difficulties [17] and academic challenges [18], cognitive training may be a suitable treatment to target multiple areas of their functioning. However, implementing cognitive training interventions and accurately assessing skills in this population presents many challenges.

Students with significant intellectual challenges are more likely to show deficits in *access skills*. These skills, such as verbal expression and comprehension, are required for a veridical estimation of participants’ cognitive ability and to remain on-task during assessments. As such, deficits in access skills have been associated with decreased assessment reliability in students with intellectual challenges [19]. Deficits in access skills, in addition to behavioral and motivational difficulties, may also affect their ability to engage with interventions. Nonetheless, recent studies have yielded positive outcomes from cognitive training programs in participants with intellectual challenges [20–22]. Collectively, these studies provide information regarding the characteristics to consider when designing and conducting an effective cognitive-training program appropriate for individuals with intellectual challenges, and include the selection of a task that (i) is accessible to the participants given their specific characteristics (e.g., requires minimal verbal language demands) and (ii) adapts to the participant’s cognitive capability.

One intervention defined by such characteristics is the NeuroTracker, a novel cognitive training program demonstrated to benefit students with developmental disabilities by resulting in improvements for both attention (near-transfer) and other related cognitive domains (far-transfer) after training [23–25]. More specifically, repeated practice on the NeuroTracker task has demonstrated the potential to benefit performance on a separate measure [25] of attention and to reduce post-concussion symptoms [24]. The NeuroTracker’s effectiveness with individuals with developmental disabilities can be attributed to three of its core characteristics. First, the task is a modernized iteration of the traditional Multiple Object-Tracking (MOT) task, which is characterized as a robust and accurate measure of selective, distributed, sustained, and dynamic attention [26, 27]. Second, the computer-based task is non-verbal and conceptually simple in nature; it involves visually tracking a subset of objects while ignoring substantially identical distractor objects over a short period of time. Third, the task’s level of difficulty adapts to the participants capability providing the ideal balance between challenge and skill level. These three characteristics (i.e., accuracy, accessibility, and adaptability) have demonstrated feasibility and appropriateness to train attention using NeuroTracker for children and adolescents with (1) different developmental disabilities with cognitive functioning between one and two standard deviations below population average [25] and (2) mild traumatic brain injury [23, 24]. However, the appropriateness of NeuroTracker training for individuals with extremely low IQ and the potential of these training benefits translating to academics remain unknown.

The aim of this study is to investigate the feasibility of successfully implementing a classroom-based study to assess the efficacy of NeuroTracker training program for individuals with cognitive functioning in the extremely low range (i.e., between 2 and 3 standard deviations below the population mean). Three feasibility objectives which aim to assess the processes and scientific parameters related to studying the effects of the intervention in this population were identified based on general guidelines for conducting feasibility studies [1]. First, we assessed the feasibility of implementing a classroom-based cognitive training study with this population by collecting data on (1) willingness to consent to participation, (2) rates of retention from baseline to post-intervention assessments, and the (3) appropriateness of inclusion criteria. We hypothesized that outside exclusion related to not meeting inclusion criteria, refusal to participate and attrition would be low. Second, we assessed the feasibility of using the selected intervention (NeuroTracker) and control task with this population by collecting data on adherence to the training regimen and performance across 15 training sessions over 5 weeks for both NeuroTracker and active control groups. It was hypothesized that the characteristics of the tasks (i.e., non-verbal, adaptable) and controlled school environment would result in high levels of adherence. The last objective was to assess the feasibility of using clinically validated instruments of math and reading performance as outcome measures to assess transfer. Specifically, the accessibility of the measures (i.e., distribution of scores and variance at baseline) and changes in pre- to post-test performance were examined to achieve the aforementioned objective. Given the wide range of difficulty and different types of scores offered by the selected measures, it was hypothesized that these standardized measures can be a suitable for this population.

## Methods

### Participants

#### Recruitment

All adolescent participants, between the ages of 13 and 17, were recruited through the Summit Centre for Research, Education and Training (SCERT), the research arm of Summit School in Montreal Canada, which serves as is a platform for streamlining its research projects for its students with developmental disabilities that result in cognitive, social, behavioral, or adaptive difficulties. The Research Ethics Board of McGill University approved the study protocol. Seventy-nine (N = 79) information packages and consent forms were sent to the parents of adolescents of 7 classrooms that were suggested by school personnel based on initial criteria (i.e., adolescents with low cognitive functioning).

#### Inclusionary/exclusionary criteria

Once parental consent was obtained, a screening assessment was conducted with each participant to obtain and estimate of their reading and math skills, and their ability to engage with examiners in the context of standardized administration. Eligibility criteria included participants: (i) with no personal or family history of a seizure disorder (e.g., epilepsy), (ii) with no conditions that would affect their vision, (iii) and characterized as having emergent reading skills (i.e., be able to at least read one word on the Word Reading task on the WIAT-III) and math skills (i.e., be able to answer at least one on the Math Fluency Addition task on the WIAT-III; measures described below). Participants’ visual acuity was assessed using Directional ‘E’ and ‘C’ near visual acuity charts, and stereopsis was assessed using the RANDOT Stereotest.

**Fig. 1:**
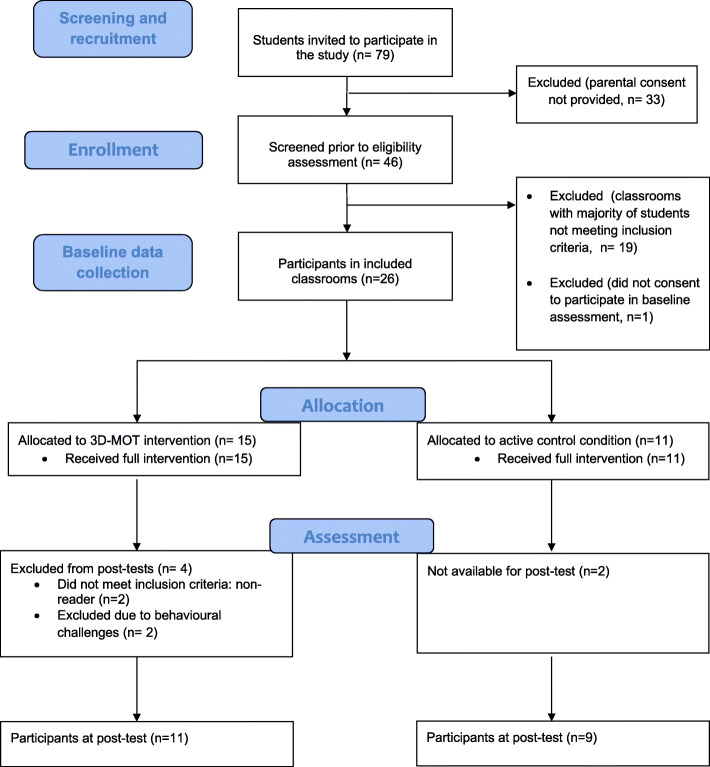
Progression of participants from initial recruitment to post-test

Additionally, participants who displayed severe behavioral difficulties during the baseline assessment (i.e., self-harm and refusal to participate) were excluded from post-intervention assessments. Following baseline assessment, three participating classrooms where a strong majority of participants did not meet inclusionary criteria were excluded from the study. Figure 1 outlines the progression of participants from screening to post-test using a Consort-style presentation.

#### Assignment to conditions

Participants meeting inclusionary criteria were distributed across four classrooms. Each participating classroom was assigned to either the treatment condition (i.e., NeuroTracker) or the active control condition (i.e., Google Chrome Dino).

### Cognitive training tasks

#### NeuroTracker (treatment)

**Fig. 2:**
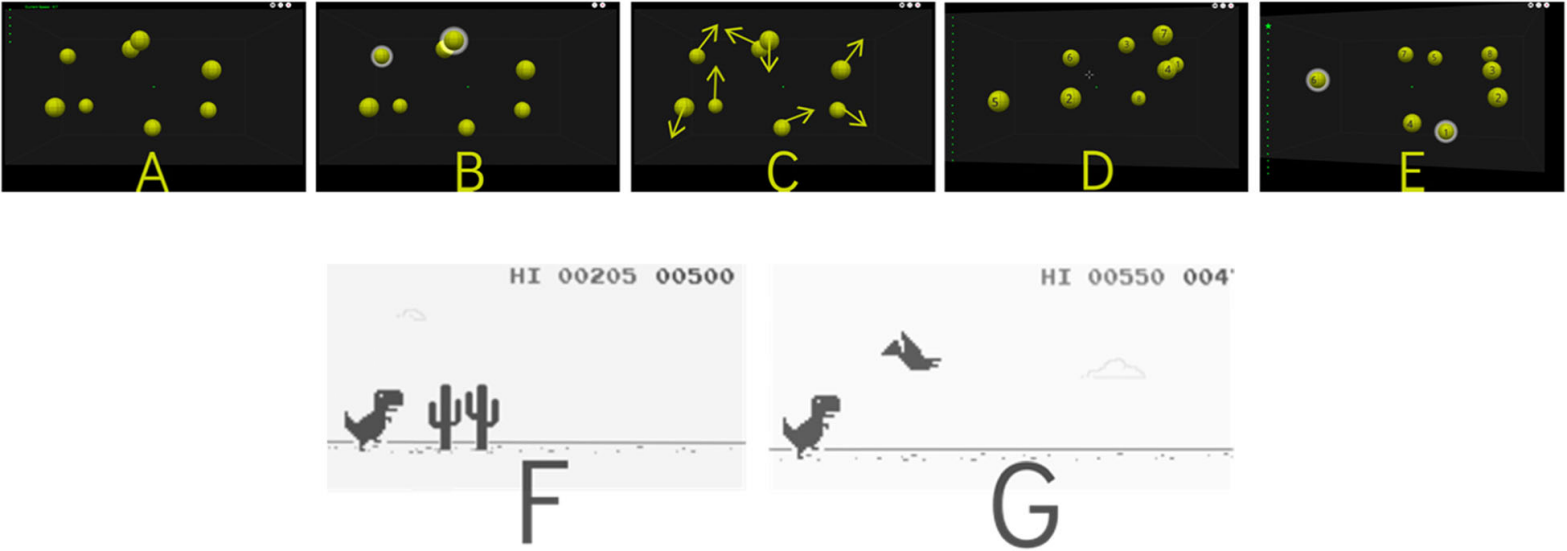
NeuroTracker and Google Chrome Dino Trial Procedures. Note. NeuroTracker trial presented across its five segments. **A** All 8 identical spheres presented within the 3D space. **B** Target spheres are highlighted with white halo. **C** Spheres are deselected and move about the 3D space for a period of time. **D** Participant indicates which spheres they were asked to track. **E** The correct spheres are highlighted providing feedback to the participant. The Google Chrome Dino trial involved. **F** Jumping over obstacles, such as trees or **G** inhibiting this behavior when jumping coincided with colliding with other obstacles, such as birds

A typical NeuroTracker training trial consisted of five key segments: presentation, identification, tracking, response, feedback (see Fig. 2A–E). During a trial, the participant was required to visually track target objects (i.e. spheres) among a number of indistinguishable distractor objects for a short period of time. Trial speed adapted to the participants capability based on their performance in the preceding trial. For instance, correctly identifying all target items increases subsequent trial speed whereas incorrectly identifying all target items decreases subsequent trial speed. In the first training session, all participants were required to track 2 spheres for 6 seconds at a predetermined speed. Training was also adapted to the participants’ capabilities by changing the difficulty level (i.e., number of targets the participants have to track (from 1 to 4) and how long they have to track them (from 4 to 8 s) from session to session based on previous performance.

#### Google Chrome Dino (active control)

Concurrent with NeuroTracker training, participants in the active control condition trained on Google Chrome Dino (GCD), which was presented on a computerized tablet. The task’s objective is to guide the dinosaur through a series of obstacles encountered on a linear path (see Fig. 2F, G).

### Procedure

#### Intervention

At randomly scheduled 20-min periods throughout the school-day, a team of research assistants entered the classroom, calibrated the tablets, and administered the respective task to participants in either the treatment or active control conditions. Participants’ training on the NeuroTracker task was provided an Apple iPad tablet and anaglyph glasses to be able to perceive the stimuli in three dimensions. For each training session, participants completed a total of 40 trials (2 blocks of 20 trials) in two consecutive blocks each lasting 5 min for a total of 10 min. Participants training on the GCD task completed their daily training session on an Apple iPad Tablet.

Training sessions occurred 3 times a week over a period of 5 weeks, for a total of 15 in-class training sessions. If necessary, additional group sessions were added at the end of the 5th training week, for participants who had missed training sessions (e.g., participants who were absent from school during a training session); missing sessions were conducted on separate calendar days and as a group activity to comply with conditions maintained throughout training. A 2:1 participant to research assistant ratio was kept throughout the training intervention to facilitate administration.

### Measures

Standardized cognitive- and academic-based assessments were conducted individually by research assistants who were graduate students who had previous training in the administration of standardized cognitive, attention, and academic measures. Testing was conducted in a designated and quiet assessment room at the SCERT (on-site) during school hours. Screening and baseline assessments were administered across two 30-min sessions. The same procedure was used to administer the math and reading measures at post-test.

#### Demographic measures

##### Wechsler Abbreviated Scale of Intelligence–Second Edition (WASI-II)

General cognitive functioning was assessed using the WASI-II [28].The four-subtest version, which consists of two subtests that involve verbal skills (Verbal Comprehension Index) and two subtests that assess non-verbal reasoning skills (Perceptual Reasoning Index) was administered. The Verbal and Performance IQ are used to provide a general estimate of intelligence or Full-Scale IQ (FSIQ).

##### Conners’ Continuous Performance Test–Third Edition (CPT 3)

The CPT 3 was used as a measure of attention [29]. The CPT 3 is a computer-based task during which participants are required to respond to letters flashed on the screen (by pressing the spacebar) as quickly and accurately as possible, but to inhibit their responses when for the letter “X”. The d’ score, a measure of detectability (or the ability to distinguish between targets and non-targets) was used as the primary outcome measure and was recorded in normalized *t* scores.

#### Program adherence measures

Program feasibility was evaluated by collecting data on adherence during sessions. For NeuroTracker training, adherence was defined as completing all forty trials within a daily training session. Of note, participants had to actively engage with the task during each trial (i.e., select a sphere on the screen by touching it) to move on to the next trial. The level of difficulty in the daily training session was also recorded as a measure of task progression throughout the training regimen. For the Google Chrome Dino control task, adherence was defined as obtaining a score greater than the minimum score within a daily training session. Data on performance (i.e., the longest distanced travelled in the current training session) during each session was also recorded to explore progress over time.

#### Outcome measures

Although evaluation of outcome to targeted domains of math and reading was not the focus of this feasibility study, academic assessments were conducted pre- and post-intervention to investigate characteristics of outcome measures. The Word Reading, Reading Comprehension, and the Math Fluency Addition, and Math Fluency Subtraction subtests of the Wechsler Individual Achievement Test, Third Edition (WIAT-III; 30) were used to assess academic skills. The WIAT-III is an individually administered norm-referenced, reliable standardized test assessing multiple aspects of academic achievement in examinees of ages 4 to 50. The norm sample included 1.1% children with ID.

##### Reading

During the Word Reading and Comprehension subtests, participants were required to read aloud a list of words of increasing difficulty presented on a page, and read passages and answer questions on its content, respectively. Participants read a set of three passages selected based on their grade level. For both subtests, the difficulty of items ranges from a grade 1 level through a grade 12 level.

##### Mathematics

During the Math Fluency Addition and Subtraction subtests, participants required to complete as many addition or subtraction problems as they could within a 60-s limit. The problems were presented visually to the participants in a booklet in which they had to write their answers. For both subtests, the difficulty of items ranges from a grade 1 level through a grade 12 level.

Raw scores, standardized scores, and growth scale values (GSV) were recorded for each subtest. Standardized scores describe participants’ performance relative to a normative sample. GSVs are based on raw scores, are sample-independent, and describe participants’ absolute level of performance on an equal interval scale. The authors of the WIAT-III recommend using the GSVs for purpose of tracking changes in performance over time [30].

### Study timeline

Participants were recruited over a 12-week period. Screening and baseline assessments were conducted over a 10-week period. All groups began training in the same week for a period of 5 weeks. Additional sessions for participants who missed a session were all conducted within a week following this period. All post-intervention assessments were completed over a 2-week period immediately following training.

## Results

### Recruitment and retention rate

Consent for participation in the study (including random allocation to intervention or control group) was obtained from the parents of 46 (n_male_ = 35; n_female_ = 11) of 79 adolescents invited to participate in the cognitive training study (58%). The four enrolled classrooms included 26 (n_male_ = 22; n_female_ = 4) of the 46 participants who had provided consent (56%). Based on information provided by the school, 42% of participants presented a diagnosis of autism spectrum disorder (ASD), 15% had a diagnosis of attention deficit hyperactivity disorder (ADHD), and 11% had Down syndrome. Table 1 presents the demographic information and baseline performance of the 26 enrolled participants.

**Table 1:** Means and standard deviations for demographic and outcome measures by condition

	Treatment (n = 11)			Active control (n = 9)			Excluded (n = 6)		
	M	SD	R	M	SD	R	M	SD	R
**Age**	15.86	0.68	15–17	15.52	0.95	13.7–17.1	15.27	0.86	14.2–16.8
**FSIQ**	59.73	16.19	40–86	50.50	7.45	40–60	52.50	12.37	40–72
**VCI**	55.27	11.56	45–82	51.88	10.44	45–69	47.50	6.12	45–60
**PRI**	69.45	22.41	45–111	53.62	6.32	45–66	62.83	17.29	45–89
**CPT-3 – d’**	62.82	10.54	44–75	69.44	4.48	61–74	67.33	2.160	64–70
**Pre-WIAT WR**	59.09	16.78	40–85	48.44	10.67	40–65			
**Pre-WIAT RC**	51.45	10.80	40–70	44.56	7.07	40–59			
**Pre-WIAT FA**	62.55	21.78	40-106	43.56	7.88	40-64			
**Pre-WIAT FS**	53.73	16.08	41–87	42.89	2.03	41–47			

*Note.* Statistics presented for demographic variables of (i) intelligence as measured by the WASI-II via Full-Scale IQ (FSIQ), Verbal Comprehension Index (VCI), and Perceptual Reasoning Index (PRI); and (ii) attention as measured by the CPT-3 d’ t score. Baseline performance on the academic outcomes was measured by the WIAT-III via Word Reading (WR), Reading Comprehension (RC), Fluency-Additions (FA), and Fluency-Subtractions (FS)

Figure 1 outlines the progression of participants from recruitment to post-test data collection. All participants successfully completed tests of visual acuity and stereopsis and thus, no participants were excluded from the study for reasons related to vision.

All 26 participants took part in baseline assessments and cognitive training tasks (NeuroTracker or GCD). Based on baseline performance assessment, two participants were excluded from post-intervention assessments because they did not meet inclusion criteria (i.e., they were considered non-readers). Of the remaining 24 participants meeting inclusion criteria, two additional participants were excluded from post-intervention assessments due to behavioral challenges (i.e., self-harm and refusal to participate during the baseline assessment) and two participants from the control group were not available for post-intervention assessments. Complete pre-training and post-training data were completed by 20 of the 24 participants meeting inclusion criteria, a retention rate from pre- through to post-test of 83%.

### Adherence/feasibility of the intervention

**Fig. 3:**
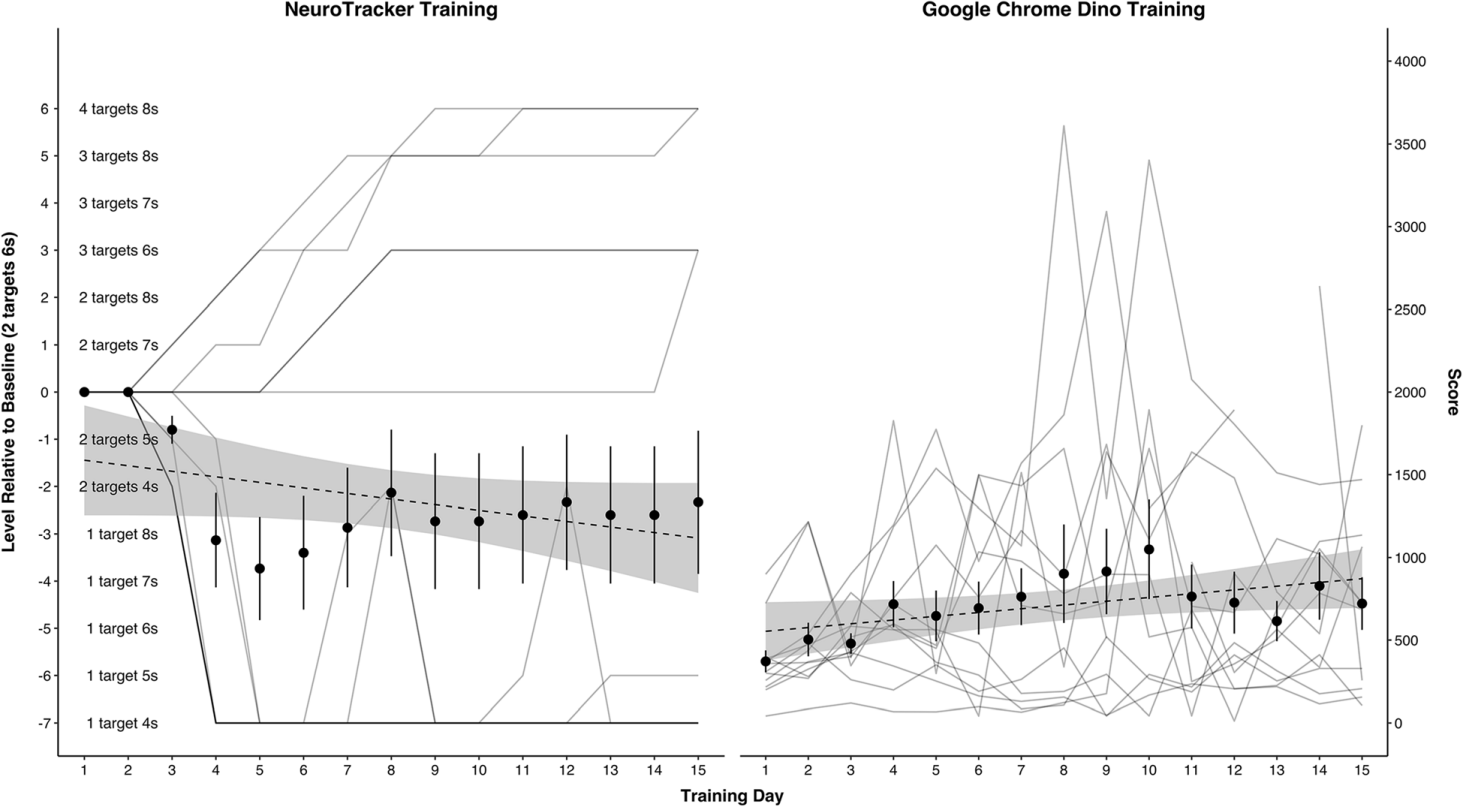
*A*dherence to treatment and active control conditions. *Note.* The two graphs present the mean and the standard error of performance in addition to a line trace for each participant. The graph on the left side illustrates NeuroTracker training across 15 training days. Performance is defined as the level of difficulty completed relative to baseline: tracking 2 targets for 6 s. The levels of difficulty range from 7 levels below the baseline level: tracking 1 target for 4 s; to 6 levels above baseline: 4 targets for 8 s. The graph on the right depicts GCD training across 15 training days. GCD training was defined as the highest score achieved in that training session; that is, the longest course run by the dinosaur

All participants in the NeuroTracker condition completed 40 trials during each of the 15 training session, indicating 100% adherence to the training program. Data was also collected on the level of difficulty of the task during each training session to explore participants’ progress on the task across the training program (see Fig. 3). When considering the average performance for all participants, the level of difficulty of the task adapted to the participants capability after the first three sessions. Overall, there was a slight increase in the average performance of participants during each session throughout the remaining sessions, as demonstrated by the trendline in Fig. 3. This suggests that on average, participants progressed through the cognitive training program that adapted to their capability over time. Analysis of individual results demonstrates that approximately half of the participants (47%) moved to higher levels of difficulty relative to baseline. The other half (53%) trained on the lowest level of difficulty throughout most of the training sessions (see left graph in Fig. 3).

Participants training with *GCD* also demonstrated perfect adherence to their training condition as evidenced by no missing data on the task for all participants. Data on performance (i.e., the longest distanced travelled in the current training session) during each session was also recorded to explore progress over time. This improvement was illustrated by the positive trendline of GCD scores across training days.

### Baseline results

Table 1 outlines the mean Full-Scale Wechsler IQ, CPT-3 performance and baseline Standard Scores on the reading and math measures for participants in both groups. To account for any preconceived imbalance between experimental conditions, we conduct independent samples t tests across measures of intelligence and attention, as well as all targeted outcomes. No statistically detectable difference between conditions was found for either intelligence: (i.e., WASI-II FSIQ; *M*_*NeuroTracker*_ *=* 59.73*,* 95% CI [50.16, 69.30]; *M*_*GCD*_ *=* 50.50*,* 95% CI [45.64, 55.36]): *t*(18) = 1.49, *p* = .153, Cohen’s *d* = 0.70, 95% confidence interval (CI) = [− 0.25, 1.57] CPT-3 performance (*M*_*NeuroTracker*_ *=* 62.82*,* 95% CI [56.59, 69.05]; *M*_*GCD*_ *=* 69.44, 95% CI [66.52, 72.37]): *t*(18) = − 1.89, *p* = .075, *d* = − 0.89, 95% CI = [− 1.76, 0.09]. Furthermore, there was no statistically detectable differences on either of the WIAT-III reading subtest measures: Word Reading (*M*_*NeuroTracker*_ *=* 422.27, 95% CI [348.68, 495.86]; *M*_*GCD*_ *=* 334.67*,* 95% CI [264.83, 404.50]): *t*(18) = 1.65, *p* = .117, *d* = 0.78, 95% CI = [− 0.18, 1.64]; Reading Comprehension (*M*_*NeuroTracker*_ *=* 414.64*,* 95% CI [390.59, 438.68]; *M*_*GCD*_ *=* 391.78, 95% CI [371.56, 411.99]): *t*(18) = 1.39, *p* = .183, *d* = 0.65, 95% CI = [− 0.29, 1.52]. However, baseline differences in the Math Fluency Additions (*M*_*NeuroTracker*_ *=* 462.63, 95% CI [379.68, 545.60]; *M*_*GCD*_ *=* 350.44*,* 95% CI 315.92, 384.97]): *t*(18) = 2.68, *p* = .015, *d* = 1.27, 95% CI = [0.23, 2.16], and Subtractions tasks (*M*_*NeuroTracker*_ *=* 389.55, 95% CI [336.18, 442.92]; *M*_*GCD*_ *=* 325.11, 95% CI [309.28, 338.94]): *t*(18) = 2.21, *p* = .040, *d* = 1.04, 95% CI = [0.04, 1.92] were found.

### Performance on outcome measures

**Fig. 4:**
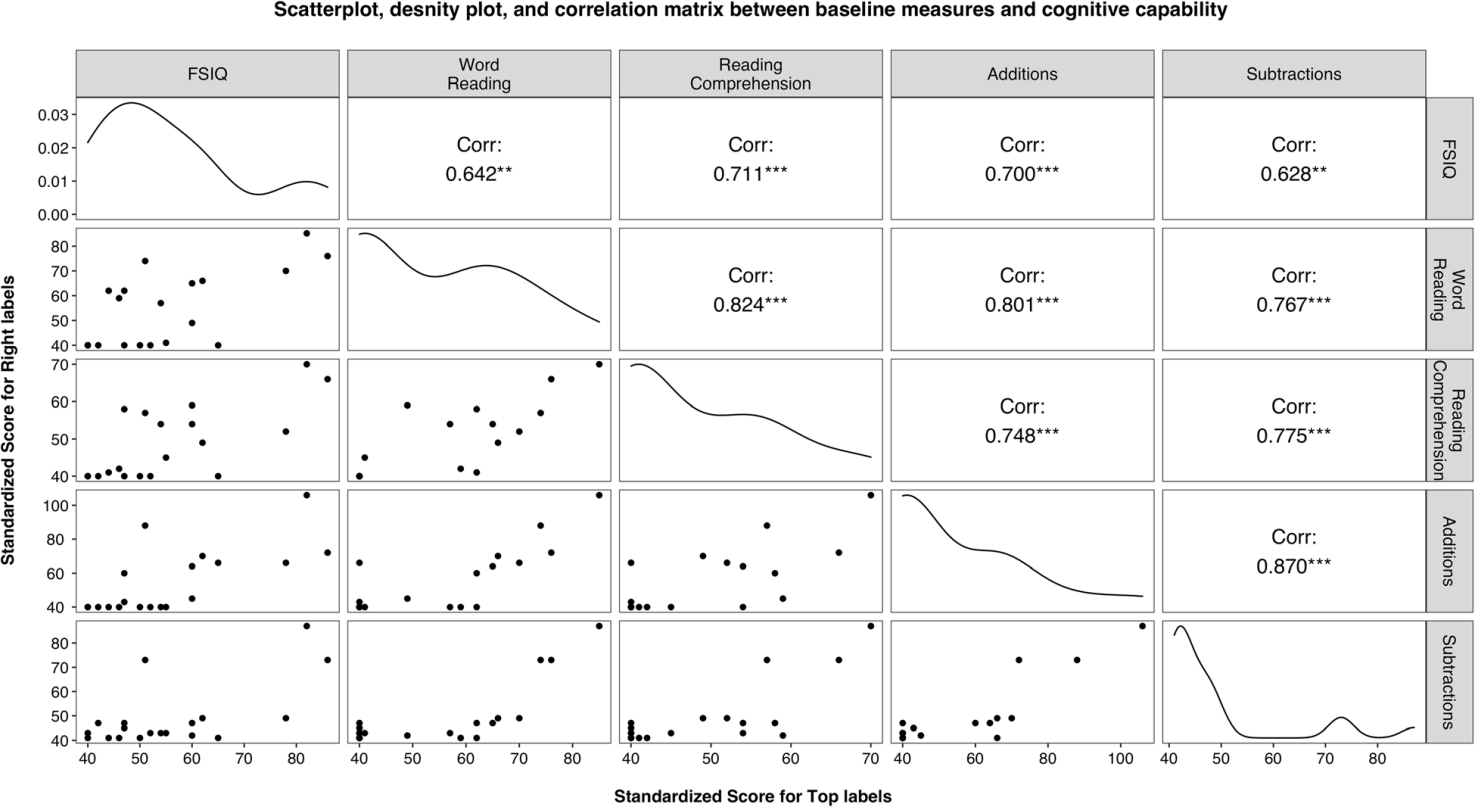
*S*catterplot, correlation matrix, and density plot between baseline measures and cognitive capability. *Note.* Scatterplot matrix between academic outcome measures and the WASI-II FSIQ score (i.e., presented as a proxy for cognitive capability) is presented on the bottom half. A density plot illustrating the distribution of scores on FSIQ and targeted outcome measures is presented on the diagonal. The correlation matrix between aforementioned variables is presented on the top half diagonal. ** p < .01, *** *p* < .001

The appropriateness of the targeted academic outcome measures for their recruited sample was tested by examining whether performance on the outcome measures co-varied with cognitive capability (i.e., FSIQ). The scatterplot, density plots, and correlation matrix in Fig. 4 illustrate the variability between FSIQ and the academic outcome measures as well as the variability within all academic outcome measures. Although the sample performed at the lower tails of the distribution across the four academic outcome measures, there was some degree of covariability between cognitive capability and the targeted outcome measures. That is, correlations ranged from .628 to .711 and scores on both measures were not clustered at the measures’ floor.

### Exploratory statistical analysis of academic assessment outcomes

**Fig. 5:**
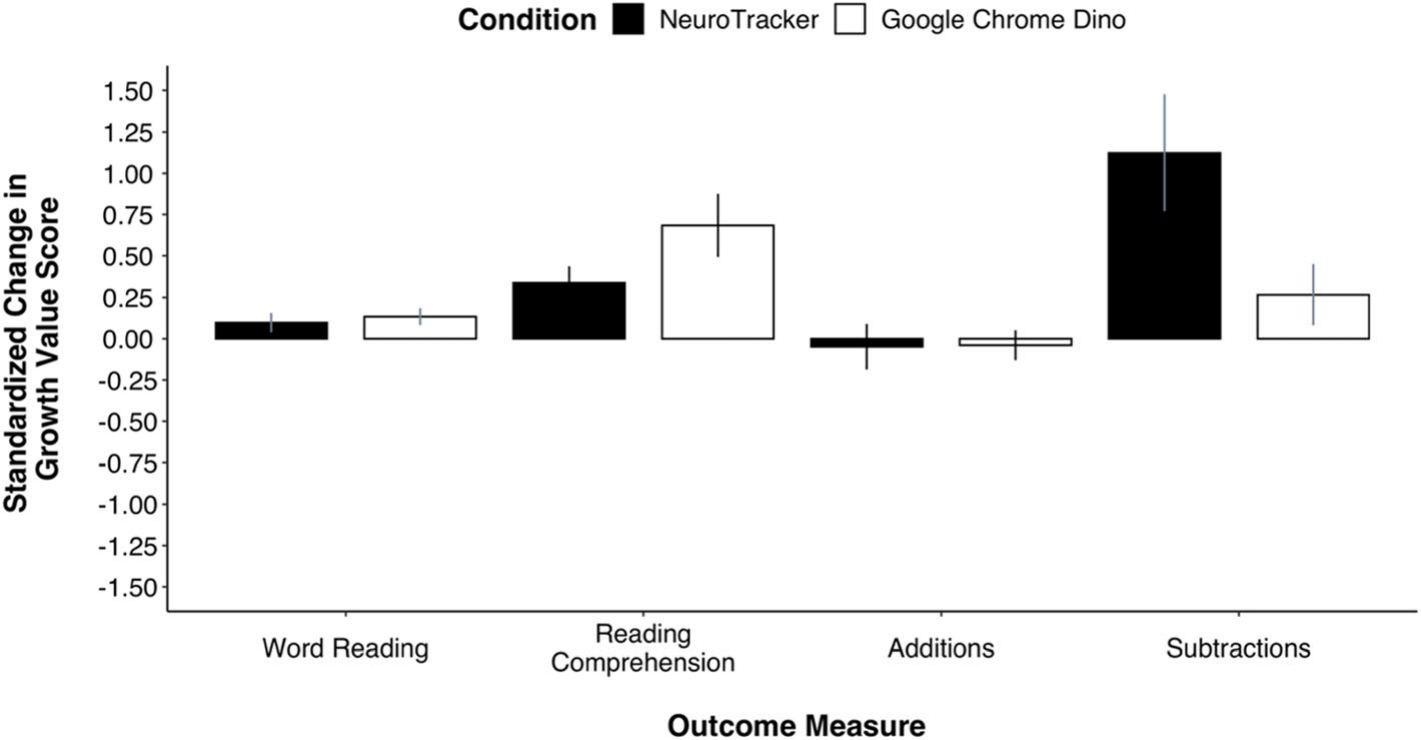
*S*tandardized change in targeted academic outcome measures by condition. *Note.* The standardized change in growth value score for each targeted outcome (i.e., reading and math) includes the standard deviation for both conditions as the denominator.

Table 2 presents the GSV scores on the reading and math measures at pre- and post-test for each condition. Eight separate one sample t tests (i.e., Bonferroni corrected alpha = .006) were conducted to examine change in performance in the post-test from the pre-test on the four targeted academic measures from statistical analyses (i.e., one-sample t test per outcome measure per condition). For consistency, we report 95% confidence intervals for all estimates; however, statistical detectability was adjudicated using the Bonferroni corrected alpha. As shown in Fig. 5, the standardized change in standard score in the post-test from the pre-test subtest was not statistically different than 0 for either group for the Math Fluency Addition task (NeuroTracker (*M*_*D*_ *=* − 0.05, 95% CI [− 0.32, 0.22]): *t*(10) = − 0.35, *p* = .733, *d* = − 0.11, 95% CI [− 0.70, 0.49]; GCD (*M*_*D*_ *=* − 0.04*,* 95% CI [− 0.22, 0.14]): *t*(8) = − 0.43, *p* = .676, *d* = − 0.14, 95% CI [− 0.80, 0.52]), the Math Fluency Subtraction task (NeuroTracker (*M*_*D*_ *=* 1.12, 95% CI [0.43, 1.82]): *t*(10) = 3.18, *p* = .010, *d* = 0.96, 95% CI 0.22, 1.67]; GCD (*M*_*D*_ *=* 0.27, 95% CI [− 0.09, 0.63]): *t*(8) = 1.44, *p* = .187, *d* = 0.48, 95% CI [− 0.22, 1.16]), and the Word Reading task (NeuroTracker (*M*_*D*_ *=* 0.10*,* 95% CI [− 0.02, 0.21]): *t*(10) = 1.67, *p* = .127, *d* = .50, 95% CI [− 0.14, 1.12]; GCD (*M*_*D*_ *=* 0.13, 95% CI [0.03, 0.23]): *t*(8) = 2.62, *p* = .031, *d* = .87, 95% CI [0.08, 1.63]). For Reading Comprehension, there was a statistically detectable difference from 0 for those training on NeuroTracker (*M*_*D*_ *=* 0.34, 95% CI [0.14, 0.53]): *t*(10) = 3.49, *p* = .006, *d* = 1.05, 95% CI [0.29, 1.78]; however, there was no statistically significant change GCD (*M*_*D*_ *=* 0.68, 95% CI [0.31, 1.06]) *t*(8) = 3.57, *p* = .007, *d* = 1.19, 95% CI [0.30, 2.04].

**Table 2:** Means and standard deviations for outcome measures at pre and post-test by condition

	Treatment (n = 11)				Active control (n = 9)			
	Baseline		Post-test		Baseline		Post-test	
	M	SD	M	SD	M	SD	M	SD
**WIAT WR**	422.27	124.53	435.00	127.38	334.67	106.89	352.11	111.48
**WIAT RC**	414.64	40.69	426.55	40.28	391.78	30.94	415.78	32.03
**WIAT FA**	462.64	140.38	455.36	115.65	350.44	52.84	344.56	68.85
**WIAT FS**	389.55	90.31	486.73	161.10	324.11	22.70	347.11	48.07

*Note.* Pre- and post-test growth scale value scores to assess appropriateness of transfer to targeted academic outcomes were measured by the WIAT-III via Word Reading (WR), Reading Comprehension (RC), Fluency-Additions (FA), and Fluency-Subtractions (FS). A higher score indicates better performance

## Discussion

The present study assessed the feasibility of implementing a study to assess the effects of a classroom-based attention training intervention (NeuroTracker) targeting academic skills in adolescents diagnosed with developmental disabilities and IQs in the extremely low range. The evaluation of the feasibility of such an intervention included the three following objectives: (1) assessing the appropriateness of classroom-based cognitive training program for the population, (2) assessing the appropriateness of using the NeuroTracker and active control task with this population, (3) assessing the validity of using standardised measures to assess far-transfer effects of the intervention on academic performance. The recruitment rate and low attrition demonstrate the feasibility of conducting a cognitive training study in a school setting. In addition, our results demonstrate that all participants in the treatment condition adhered to the training protocol, which suggests that using this intervention is feasible with this population. Finally, it was found that despite some limitations due to floor effects, the standardized reading and math tasks were sensitive to individual differences in performance.

### Feasibility of implementing a cognitive training program

Our first objective assessed the feasibility of implementing a classroom-based cognitive training program with participants with extremely low cognitive functioning. Given the relatively low incidence of this type of cognitive profile, it was critical to determine if a sufficient proportion of parents, teachers, and adolescents with intellectual challenges are interested in the implementation of cognitive training interventions in their classroom. Our results suggest that slightly over half (58%) of families contacted consented to participation in a school-based cognitive training study and that adolescents generally consent to receiving the intervention. To our knowledge, this is the first study documenting willingness to participate in a cognitive training intervention study for this population. While our results are limited to a specific setting (i.e., a private school providing specialized services), they confirm that school principals, teachers, and parents of adolescents with intellectual challenges perceive the need for interventions that target deficits in cognitive abilities such as attention. Our findings also provide valuable information regarding the recruitment rate that can be expected in a larger-scale study.

In addition to initial interest, it was also critical to assess attrition, as it can pose a significant obstacle towards evaluating the efficacy of a cognitive training paradigm [31]. Outside of participants not meeting inclusion criteria, the retention rate from baseline to post-intervention assessments (83%) was similar to previous cognitive training studies conducted with participants with intellectual challenges [20, 22]. This low attrition rate suggests that the results of the study can be assessed fairly as they were not biased by the factors that can lead to attrition (e.g., motivation, harm, perceived cost-benefit). We hypothesize that the school-based nature of this study could have contributed to the high retention rates as it facilitated access the participants and monitoring throughout the study. The low attrition rate also suggests that our inclusion criteria were effective in identifying the participants that would be able to complete all stages of the study.

With regard to inclusion criteria, our study addressed a gap in the current cognitive training literature, which typically excludes participants with intellectual functioning below the average range (see [14–16] for examples). Our results are consistent with those of the few previous studies which show that it is feasible to assess the effects of cognitive training in students with similar cognitive profiles [20–22]. While our eligibility criteria were similar to the one used in those studies, participants who were likely to meet inclusion criteria prior to recruitment were not selected. As a result, 3 of the 7 recruited classrooms (44% of recruited participants) were excluded following the screening assessment due to not meeting inclusion criteria. The academic performance of most of the participants in the excluded classrooms could not be assessed fairly as they did not possess the access skills necessary to engage with standardized tasks (e.g., were non-verbal, had significant difficulties understanding questions and instructions). Therefore, our findings expand on the current cognitive training literature by indicating that while we can successfully implement cognitive training study with individuals with extremely low IQ, a subset of this population (i.e., individuals functioning at the lower end of the distribution) lacks the skills necessary to participate in such a study.

### Feasibility of using NeuroTracker training for adolescents with extremely low IQs

We hypothesized that due to its core characteriscs (i.e., accuracy, accessibility, and adaptability), the NeuroTracker would be a suitable intervention to target academic difficulties in this population. Given the strong relationship between attention and academic performance [8], an intervention which accurately targets attention such as the NeuroTracker could be particularly beneficial for the population targeted in this study. With regard to accessibility, the complete adherence to the intervention indicates that this intervention is accessible to adolescents with extremely low cognitive functioning, expanding on findings of a study conducted with adolescents with cognitive functioning 1 to 2 standard deviations below average [25]. Our results also demonstrate that the program can be implemented as a group intervention and that one-to-one supervision is not necessary for successful implementation, further demonstrating the accessibility of the task.

Another caracteristic of the NeuroTracker which we hyposthesized would make it suitable for this population is its adaptability. Previous research has highlighted that adapting the task difficulty to a participant’s capacity is a valuable feature of effective cognitive training interventions [32]. The NeuroTracker is highly adaptable, as it modifies task difficulty based on participants’ performance in three different ways (i.e., by adapting speed, duration of the task, and the number of targets). It remains unclear whether the task was adaptive enough to meet the needs of all participants, as data collected on the difficulty level attained during each session suggest that about half of the participants showed progress on the task throughout the 5 weeks, while the other half trained on the lowest level of difficulty throughout most sessions. A similar variability in training performance was reported by [22], who examined the effects of a computerized cognitive training task targetting non-verbal reasoning and working memory in children with ID. Furture studies will be necessary to examine whether these participants were making progress within the lowest level, or if they were simply not engaging with the task because it was above their baseline capacity. Nonetheless, high adherence on the NeuroTracker increases the likelyhood that particpants will benefit from this intervention.

The adaptive nature of the NeuroTracker is based on factors that influence an individual performance on the Multiple Object-Tracking (MOT) task, the psychophysical task that the NeuroTracker is based on. Previous research examining MOT capability has demonstrated that manipulating the paradigm’s task demands (i.e., similar to those manipulated here to adapt to the participant’s capability) quantifies the attentional resources required to successfully complete the trial [27, 33]. Thus, any change in either the number of target items, the speed of all objects, and/or tracking duration is directly associated with the task’s attentional demands or level of difficulty.

One critical advantage of using the NeuroTracker with students with extremely low IQ is its ability to adapt to the participants’ capability by increasing or decreasing trial speed, depending on correct or incorrect responses, respectively. The importance of adaptability has been demonstrated with other cognitive training paradigms [34–36]. In fact, published meta-analyses that have concluded that adaptive cognitive training programs produced larger effects than non-adaptive programs (see [37, 38]). Nevertheless, there is a lack of research that has assessed adaptability as a variable for identifing optimal adaptive training protocols. As such, future research examining the benefits of diverse levels of adaptivity would be a significant contribution to uncover the mechanisms of NeuroTracker training as well as cognitive training at large.

### Scientific validity assessment

In addition to assessing the feasibility of using the NeuroTracker with adolescents with extremely low cognitive functioning, an important objective of this study was to assess the suitability of clinically validated academic measures in this population. Exploratory correlations between scores on the cognitive and academic measures were conducted to examine the degree of co-variability between them. The objective of these exploratory analyses was not to infer a relationship between cognitive and academic capability. In fact, there are significant violations to the statistical assumptions that preclude this conclusion; (i) the variables are not normally distributed, (i) the sample size is small, and (iii) the sample recruited represents one portion of the normal distribution (i.e., restricted range). Rather, the analyses allowed us to observe the spread of the scores and the covariance between the cognitive and academic measures. Floor effects have been identified as being an issue in reducing range and variability when using standardized measures to assess abilities in individuals with significant intellectual challenges (e.g., [39, 40]). Therefore, a large cluster at the floor of each measure would have been an indication that these measures are not an appropriate tool to detect individual differences in performance in this population. However, the spread of scores (i.e., absence of large clusters at the floor of each measure) suggest that the measures presented an adequate degree of sensitivity in detecting individual differences across both cognitive and academic domains. Slightly larger floor effects were found for the Reading Comprehension and the Math Fluency Subtraction tasks of the WIAT-III.

A closer examination of Raw Scores on these tasks also indicates that a higher proportion of participants could not complete them. About a quarter (27%) of participants meeting inclusion criteria were unable to answer any questions on the reading comprehension task. As the task’s easiest item requires reading 8 short sentences, we hypothesize that it is too advanced to assess the reading comprehension skills of emergent readers (i.e., participants who could recognize some individual words but had difficulty reading connected text). The small floor effect observed on this task could also be affected by access skills, as the task places a high demand on verbal skills (e.g., understand the examiner’s question, respond verbally). A similar proportion of participants (30%) were unable to answer any of the Math Fluency Subtraction items correctly. In this case, we hypothesize that the distribution of scores likely represents the skill level of participants rather than deficits in access skills, as the presentation of the task was similar to the Addition task, which had a higher completion rate. Nevertheless, the degree of variability presented by the values suggests that overall, the measures were representative of the participant’s academic capabilities.

Exploratory analysis of changes in academic performance before and after training was also conducted to explore whether the measures could detect changes in performance following training. We note that the objective of these analyses was not to assess the effectiveness of the NeuroTracker in improving academic performance, and that given the small sample size we do not suggest that the results demonstrate transferability of training to these academic skills. Significant changes in performance from baseline to post-intervention were found for the intervention group on the Reading Comprehension task. We suggest that these results indicate that standardized academic measures can detect individual differences in reading and math skills and potentially detect changes in performance as a function of the intervention. The use of “in-house” measures which lack reliability and real-world validity has been highlighted as a significant limitation of the current cognitive training research [1, 13]. Our results demonstrate standardized academic measures are an appropriate and feasible mean to assess change in academic performance as a result of cognitive training, even in atypically developing populations. The use of such clinically validated and highly-reliable instruments reduces sources of error and improves our ability to accurately assess the efficacy of the cognitive training program.

### Limitations

A limitation of this study was the inability to examine the performance of participants within levels of difficulty. As a result, it was not possible to assess the adaptability within the levels of difficulty. Therefore, it remains unclear whether participants that ended training at the lowest level of difficulty were representative of their capability and whether they improved within this level. Nonetheless, the data collected suggests the task presents with a wide range of adaptability which detected improvements in performance in half of participants. A further limitation was that the study’s inclusion criteria limited participation to a group of adolescents who presented emergent reading and math skills and who showed a certain ability to engage with standardized tasks (i.e., could answer questions verbally, showed minimal behaviour problems). While this limited the generalizability of our findings to adolescents who have similar characteristics, we believe this was necessary in order to evaluate the feasibility of using standardized measures to assess response to a cognitive training intervention for this underserved subpopulation. Finally, as the study did not include a follow-up assessment, we could not determine if retention rates would remain high if another assessment was conducted several weeks or months after post-test to investigate the long-term effects of the training program. The results of the post-test provide us evidence and support that a follow-up assessment would be feasible and warranted. Therefore, the inclusion of follow-up assessments would be of particular importance in future studies, as it is hypothesized that training benefits may be delayed rather than immediate [1, 13].

### Implications for future studies

The information obtained through this feasibility study suggest provided support for the feasibility of conducting studies to assess the viability of using the NeuroTracker with adolescents with significant intellectual challenges. Therefore, our findings suggest that families are interested in participating in such a study and that this population can comply with training and engage with the NeuroTracker. Results from this study provided support for the use of standardized measures to assess academic performance, while identifying limitations that should be addressed in future studies, such as selection of a reading comprehension task with easier floor items (e.g., tasks that assess reading comprehension by asking to match a word or phrase with a picture). It was also found that while participants were all selected from classrooms which group students with similar functioning levels, there was a significant variability in their progress throughout training. This emphasizes the importance for future studies to investigate the impact of individual factors (e.g., IQ, baseline attentional capacities, co-morbid diagnosis) on the response to intervention.

Objectives centered on identifying individual differences factors that predict to improvement as a function of the intervention are examined in effectiveness studies (as defined by 1). Although our current study collected such baseline and/or non-trained cognitive data, the small sample size of participants in the treatment condition (n = 15) precludes any concrete conclusions on the role of individual differences factors here. Moreover, previous research examining the efficacy of NeuroTracker found no evidence of an association between improvement on the training paradigm and improvement in the non-trained targeted outcome [25]. As such, the mechanisms underlying the translation of benefits from the training paradigm remain unclear. The findings from the current study encourage future research arch questions that explore the factors that explain differences in program efficacy (i.e., improvement) and the underlying mechanisms at play, which translate to improvement in targeted outcomes.

Finally, the optimization of treatment duration (i.e., the amount of training sessions) and/or training dosage (i.e., the amount of time spent training) remains unknown for training with NeuroTracker, as is the case throughout the field of cognitive training. While meta-analyses have highlighted that time spent in repeated practice is positively related to degree of improvement in targeted outcomes [38, 41, 42], the optimal levels of treatment duration or time per training session remains unknown. Thus, research aimed at optimizing treatment duration and dosage is of great importance and would significantly advance the field of cognitive training at large, it is best served in research uncovering the mechanisms of the cognitive training program, rather than in research assessing the feasibility to implement a cognitive training program and/or the appropriateness of the training paradigm for specific populations, as assessed here.

## Conclusions

Despite the growing litterature on cognitive training programs targetting abilities such as working memory or attention, very few protocols have been developped or validated to meet the needs of children with significant intellectual challenges, an underserved portion of the student population. The present study demonstrated the feasibility of a school-based cognitive training intervention in adolescents with extremely low cognitive functioning and diverse developmental disabilities. Whereas most cognitive training studies usually exclude participants with below-average IQs, the current study focused on participants that presented well below average cognitive functioning. Our results suggest that the NeuroTracker represents an accessible and appropriate intervention to improve attention in this population. In addition, information obtained here can have implications for designing and assessing the effectiveness of interventions in this population [1]. Another strength of this of this study was the use of standardized math and reading tasks which had documented reliability and provided clinically interpretable scores. Although two of the outcome measures were too difficult for some of the participants, we believe that the measures were sensitive enough to reflect variability in performance at baseline and potentially assess far-transfer effects. Given these considerations, we recommend the continuation of effectiveness studies on cognitive training in children and adolescents with extremely low cognitive functioning. Overall, strengthening the knowledge of the feasiblity of implementing an efficacy and/or effectiveness study for typically underserved populations, as well as assessing the appropriateness of targeted outcome measures extends the knowledge in the field of cognitive training. Additionally, further validating promising cognitive training paradigms has implications for parents of children with developmental disabilities, teachers, and education professionals, providing an alternative or supplemental approach towards improving academics and other-realted cognitive deficits.

## Data Availability

The datasets used and/or analyzed during the current study are available from the corresponding author on reasonable request.

## References

[1] Green CS, Bavelier D, Kramer AF, Vinogradov S, Ansorge U, Ball KK, et al. Improving methodological standards in behavioral interventions for cognitive enhancement. J Cogn Enhanc. 2019 [cited 2019 Feb 12]; Available from:. 10.1007/s41465-018-0115-y.

[2] Au J, Buschkuehl M, Duncan GJ, Jaeggi SM (2016). There is no convincing evidence that working memory training is NOT effective: A reply to Melby-Lerväg and Hulme (2015). Psychon Bull Rev.

[3] Sonuga-Barke EJS, Brandeis D, Cortese S, Daley D, Ferrin M, Holtmann M, Stevenson J, Danckaerts M, van der Oord S, Döpfner M, Dittmann RW, Simonoff E, Zuddas A, Banaschewski T, Buitelaar J, Coghill D, Hollis C, Konofal E, Lecendreux M, Wong ICK, Sergeant J, European ADHD Guidelines Group (2013). Nonpharmacological interventions for ADHD: Systematic review and meta-analyses of randomized controlled trials of dietary and psychological treatments. AJP..

[4] Sonuga-Barke EJS, Brandeis D, Holtmann M, Cortese S (2014). Computer-based cognitive training for ADHD: a review of current evidence. Child Adolesc Psychiatr Clin N Am.

[5] Spaniol MM, Shalev L, Kossyvaki L, Mevorach C (2017). Attention training in autism as a potential approach to improving academic performance: a school-based pilot study. J Autism Dev Disord.

[6] Jones MR, Katz B, Buschkuehl M, Jaeggi SM, Shah P (2020). Exploring n-back cognitive training for children with ADHD. J Atten Disord.

[7] Shalev L, Tsal Y, Mevorach C (2007). Computerized Progressive Attentional Training (CPAT) Program: Effective direct intervention for children with ADHD. Child Neuropsychol.

[8] Romano E, Babchishin L, Pagani LS, Kohen D (2010). School readiness and later achievement: replication and extension using a nationwide Canadian survey. Dev Psychol.

[9] Cragg L, Gilmore C (2014). Skills underlying mathematics: the role of executive function in the development of mathematics proficiency. Trends Neurosci Educ.

[10] May T, Rinehart N, Wilding J, Cornish K (2013). The role of attention in the academic attainment of children with autism spectrum disorder. J Autism Dev Disord.

[11] Rabiner DL, Murray DW, Skinner AT, Malone PS (2010). A randomized trial of two promising computer-based interventions for students with attention difficulties. J Abnorm Child Psychol.

[12] Melby-Lervåg M, Redick TS, Hulme C (2016). Working memory training does not improve performance on measures of intelligence or other measures of “far transfer”: evidence from a meta-analytic review. Perspect Psychol Sci.

[13] Simons DJ, Boot WR, Charness N, Gathercole SE, Chabris CF, Hambrick DZ, Stine-Morrow EAL (2016). Do “Brain-Training” programs work?. Psychol Sci Public Interest.

[14] Ackermann S, Halfon O, Fornari E, Urben S, Bader M (2018). Cognitive Working Memory Training (CWMT) in adolescents suffering from attention-deficit/hyperactivity disorder (ADHD): a controlled trial taking into account concomitant medication effects. Psychiatry Res.

[15] Chacko A, Bedard A-CV, Marks D, Gopalan G, Feirsen N, Uderman J, Chimiklis A, Heber E, Cornwell M, Anderson L, Zwilling A, Ramon M (2018). Sequenced neurocognitive and behavioral parent training for the treatment of ADHD in school-age children. Child Neuropsychol.

[16] Orylska A, Hadwin JA, Kroemeke A, Sonuga-Barke E. A growth mixture modeling study of learning trajectories in an extended computerized working memory training programme developed for young children diagnosed with Attention-Deficit/Hyperactivity Disorder. Front Educ. 2019; [cited 2019 Jul 12];4. Available from: https://www.frontiersin.org/articles/10.3389/feduc.2019.00012/full.

[17] Antshel KM, Zhang-James Y, Wagner KE, Ledesma A, Faraone SV (2016). An update on the comorbidity of ADHD and ASD: a focus on clinical management. Expert Rev Neurother.

[18] Wei X, Blackorby J, Schiller E (2011). Growth in reading achievement of students with disabilities, ages 7 to 17. Except Child.

[19] Niebling BC, Elliott SN (2005). Testing accommodations and inclusive assessment practices. Assess Eff Interv.

[20] Bennett SJ, Holmes J, Buckley S (2013). Computerized memory training leads to sustained improvement in visuospatial short-term memory skills in children with Down syndrome. Am J Intellect Dev Disabil.

[21] Dahlin E, Nyberg L, Bäckman L, Neely AS (2008). Plasticity of executive functioning in young and older adults: Immediate training gains, transfer, and long-term maintenance. Psychol Aging.

[22] Söderqvist S, Nutley SB, Ottersen J, Grill KM, Klingberg T (2012). Computerized training of non-verbal reasoning and working memory in children with intellectual disability. Front Hum Neurosci.

[23] Corbin-Berrigan L-A, Kowalski K, Faubert J, Christie B, Gagnon I (2018). Three-dimensional multiple object tracking in the pediatric population: the NeuroTracker and its promising role in the management of mild traumatic brain injury. NeuroReport..

[24] Corbin-Berrigan L-A, Faubert J, Gagnon I (2020). Neurotracker as a potential mean of active rehabilitation in children with atypical mild traumatic brain injury recovery: a pilot safety study. Translat Sports Med.

[25] Tullo D, Guy J, Faubert J, Bertone A (2018). Training with a three-dimensional multiple object-tracking (3D-MOT) paradigm improves attention in students with a neurodevelopmental condition: a randomized controlled trial. Dev Sci.

[26] Scholl BJ (2009). What have we learned about attention from multiple-object tracking (and vice versa)?. Computation, Cognition and Pylyshyn.

[27] Tullo D, Faubert J, Bertone A (2018). The characterization of attention resource capacity and its relationship with fluid reasoning intelligence: a multiple object tracking study. Intelligence..

[28] Wechsler D (2011). Wechsler Abbreviated Scale of Intelligence–Second Edition (WASI-II).

[29] Conners KC (2014). Conners Continuous Performance Test 3rd Edition (Conners CPT 3) & Conners Continuous Auditory Test of Attention (Conners CATA): Technical Manual.

[30] Wechsler D (2009). Wechsler Individual Achievement Test.

[31] Katz B, Jones MR, Shah P, Buschkuehl M, Jaeggi SM (2016). Individual differences and motivational effects. Cognitive training: an overview of features and applications.

[32] Klingberg T (2010). Training and plasticity of working memory. Trends Cogn Sci.

[33] Meyerhoff HS, Papenmeier F. Individual differences in visual attention: a short, reliable, open-source, and multilingual test of multiple object tracking in PsychoPy. Behav Res Ther. 2020 [cited 2020 Jun 17]; Available from:. 10.3758/s13428-020-01413-4. 10.3758/s13428-020-01413-4 32495028

[34] Jaeggi SM, Buschkuehl M, Jonides J, Perrig WJ (2008). Improving fluid intelligence with training on working memory. PNAS..

[35] Jaeggi SM, Buschkuehl M, Jonides J, Shah P (2011). Short- and long-term benefits of cognitive training. PNAS..

[36] Stepankova H, Lukavsky J, Buschkuehl M, Kopecek M, Ripova D, Jaeggi SM (2014). Dose-response relationship of working memory training and improvements in fluid intelligence: a randomized controlled study in old adults. Dev Psychol.

[37] Peng P, Namkung J, Barnes M, Sun C (2016). A meta-analysis of mathematics and working memory: Moderating effects of working memory domain, type of mathematics skill, and sample characteristics. J Educ Psychol.

[38] Weicker J, Villringer A, Thöne-Otto A (2016). Can impaired working memory functioning be improved by training? A meta-analysis with a special focus on brain injured patients. Neuropsychology..

[39] Orsini A, Pezzuti L, Hulbert S (2015). Beyond the floor effect on the Wechsler Intelligence Scale for Children – 4th Ed. (WISC-IV): calculating IQ and Indexes of subjects presenting a floored pattern of results. J Intellect Disabil Res.

[40] Whitaker S, Wood C (2008). The distribution of scaled scores and possible floor effects on the WISC-III and WAIS-III. J Appl Res Intellect Disabil.

[41] Schwaighofer M, Fischer F, Bühner M (2015). Does working memory training transfer? A meta-analysis including training conditions as moderators. Educ Psychol.

[42] Scionti N, Cavallero M, Zogmaister C, Marzocchi GM. Is cognitive training effective for improving executive functions in preschoolers? A systematic review and meta-analysis. Front Psychol. 2020; [cited 2021 Feb 4];10. Available from: https://www.frontiersin.org/articles/10.3389/fpsyg.2019.02812/full?utm_source=F-AAE&utm_medium=EMLF&utm_campaign=MRK_1209521_69_Psycho_20200114_arts_A. 10.3389/fpsyg.2019.02812 PMC6965160 31998168

